# Job Maintenance through Supported Employment PLUS: A Randomized Controlled Trial

**DOI:** 10.3389/fpubh.2016.00194

**Published:** 2016-09-20

**Authors:** Nils-Torge Telle, Jörn Moock, Sandra Heuchert, Vivian Schulte, Wulf Rössler, Wolfram Kawohl

**Affiliations:** ^1^Competence Tandem Integrated Services, Innovation Incubator, Leuphana University of Lüneburg, Lüneburg, Germany; ^2^Laboratory of Neuroscience (LIM 27), Institute of Psychiatry, University of Sao Paulo, Sao Paulo, Brazil; ^3^University of Zurich, Zurich, Switzerland; ^4^Department of Psychiatry, Psychotherapy, and Psychosomatics, Center for Social Psychiatry, Psychiatric Hospital, University of Zurich, Zurich, Switzerland

**Keywords:** job maintenance, sickness absence, individual placement and support, mental health, job coaching

## Abstract

Sickness absence from work due to experienced distress and mental health issues has continuously increased over the past years in Germany. To investigate how this alarming development can be counteracted, we conducted a randomized controlled trial evaluating a job coaching intervention to maintain the working capacity of members of staff and ultimately prevent sickness absence. Our sample included *N* = 99 employees who reported mental distress due to work-related problems. The intervention group (*n* = 58) received between 8 and 12 individual job coaching sessions in which they worked with a professional job coach to reduce their mental distress. The control group (*n* = 41) received a brochure about mental distress. Data were collected before the start of the study, at the end of the job coaching intervention, and at a 3-month follow-up. These data included the number of sickness absence days as the primary outcome and questionnaire measures to assess burnout indicators, life satisfaction, and work-related experiences and behaviors. Compared with the control group, the results indicated no reduction in sickness absence in the intervention group but fewer depressive symptoms, a heightened ability of the participants to distance themselves from work, more experience of work-related success, less depletion of emotional resources, and a greater satisfaction with life when participants had received the job coaching. Thus, although we could not detect a reduction in sickness absence between the groups, job coaching was shown to be a viable intervention technique to benefit employees by contributing to re-establish their mental health. We discuss the implications of the study and outline future research.

## Introduction

Work-related mental distress can often develop into more severe forms of mental illness that require therapeutic interventions and cause longer periods of sickness absence. During the last decade, sickness absence in Germany due to experienced mental illness has increased considerably (1) and is generally considered to be a serious problem in Europe (2). Mental illness may also occur as psychological comorbidities of somatic disorders, a fact only rarely noted by most physicians (3). This circumstance may contribute to the increasing number of sickness absence days (1), which ultimately represents a temporary loss of human capital and diminished organizational productivity.

Encouraging results regarding the effectiveness of job coaching have been reported for people with mental disorders receiving individual placement and support (IPS) interventions (4–6) to reintegrate them into the job market. However, it may be more effective to prevent people with mental illness or strain from losing their jobs in the first place. In this regard, supported employment interventions are often used in clinical practice and represent an established tool to help distressed employees maintain their jobs (7). IPS follows eight core principles that are listed in Table 1. Thus, IPS-based job coaching, as a specific supported employment tool, may represent a measure that companies could introduce in order to sustain the ability of their workforce to work viably (8–10).

**Table 1:** **The practice principles of IPS Supported Employment according to the Dartmouth IPS Supported Employment Center (http://www.dartmouth.edu/~ips/page48/page79/files/ips-practice-principles-002880029.pdf)**.

IPS practice principle		Description
1	Competitive employment is the goal	Work in the competitive job market alongside with others without psychiatric disabilities is aimed for
2	IPS supported employment is integrated with treatment	Thus, an exchange of information between job coach and therapist concerning work, side effects, etc. is provided
3	Zero exclusion: eligibility is based on client choice	Regardless of symptoms or other obstacles, every person with severe mental illness who wants to work is eligible for IPS
4	Attention to client preferences	The client decides on which job to search and whether to disclose his disorder
5	Benefits counseling is important	The job coach provides guidance concerning government entitlements, such as social securities for the client
6	Rapid job search	No pre-employment assessment or training is done; a job is searched for right away
7	Systematic job development	Relationships between job coaches and employers are developed, networking is important
8	Time unlimited support	Follow-along support is continued individually as long as the client needs it

Recent meta-analytic evidence provided by Theeboom et al. (11) generally indicates the effectiveness of job coaching interventions in organizational contexts. The authors of this study report several benefits of such interventions, such as improved well-being, more effective coping and better work attitudes. More specifically, Duijts et al. (12) investigated whether preventive job coaching for employees who were previously identified as being at risk for sickness absence due to psychosocial health complaints [see Ref. (13)] could reduce sickness absence. These researchers found a positive effect of job coaching on objective sickness absence due to psychosocial health complaints in the mid-term (baseline to 8-month follow-up) but only for the intention-to-treat analyses in the long term (8–12 months and the entire year after baseline follow-up). Moreover, the authors report significant effects of job coaching in terms of an improvement in the participants’ self-rated health, less burnout exhaustion as well as fewer depressive reactions for the 1 year after the baseline follow-up period for the per-protocol analyses.

## Rationale of the Present Trial and Hypotheses

Although the present study is similar to that of Duijts et al. (12), it differs in three ways. First, the present sample includes employees who subjectively felt mentally distressed and thought that they would benefit from job coaching, as opposed to employees who were specifically identified as being “‘at risk’ for sickness absence due to psychosocial health complaints” [(12), p. 766]. Second, whereas Duijts et al. (12) conducted two three-way consultations during the job coaching intervention by including the related supervisor of the employee in the second and the last coaching sessions, we excluded any third parties and the job coaching was kept confidential between the job coach and the employee. Third, to the best of our knowledge, the present study is the first to examine the effects of job coaching in organizational contexts within a German-speaking population. Based on encouraging IPS research (5, 6) and the results reported by Duijts et al. (12), we aim to investigate (a) whether job coaching can reduce the number of sickness absence days at a 3-month follow-up and (b) whether job coaching improves the mental health of employees who feel distressed.

## Materials and Methods

### Ethics

The present study has been registered under ISRCTN02422335. It was approved by the ethics committee of Leuphana University of Lüneburg and has been conducted in accordance with the Declaration of Helsinki (14). All participants gave their written informed consent prior to commencing with the study. A study protocol (14) has been published.

### The Job Coaching Intervention

We evaluate the concept of an IPS-based job coaching intervention for mentally distressed employees. Traditional IPS has been conceptualized to reintegrate people with mental illness into the open job market with the help of a job coach. In our study, an IPS-based job coaching intervention was tested for its ability to sustain the employees’ capacity to work and support employees who felt hampered in their capacity to work due to mental distress or mental illness that they experienced. The intervention comprises employee-centered, individual job coaching with the ultimate goal of maintaining the job. The job coaching was specifically tailored toward each employee’s problems and the current individual job situation. Furthermore, it enabled the employees to help themselves. The coaching entailed working on a personal job-related problem with a specially trained job coach. During the job coaching intervention, the employees and the job coach first jointly defined personal goals, which the job coach evaluated on a regular basis to facilitate goal achievement. Examples for such goals are to establish communication with the person’s superior, to find a therapist, to make a personal plan for breaks during the working hours, and to seek and handle social interaction at the workplace. The intervention included ~8–12 coaching sessions over a timespan of ~3 months. Due to the highly individualized character of IPS, many parameters, such as length of session, are not standardized but are usually no longer than 1 h.

### Study Design

The present study was a randomized controlled trial using a one-factorial design with two groups (job coaching intervention vs. control). After having given their written informed consent, the participants were randomly assigned to either the intervention or the control group via a randomized participation slot list in blocks of 10. After a new participant was assigned a participant ID by one of the job coaches, the project coordinator was solely told the ID and assigned it to a free slot on the participation list. Thus, slots were filled anonymously with participant IDs and consecutively as participants were recruited. The intervention group received the IPS-based job coaching. Participants in the control group were given an information brochure about how to best cope with mental distress.

Data were collected from each participant at three different points in time as part of a standardized interview procedure, including different questionnaires (see Table 2): immediately after the employees were assigned to the intervention or the control group (T0), after 3 months (T1; the end of the job coaching for participants of the intervention group) and after 6 months (T2; 3-month follow-up). The interviews were conducted by a person who had not interacted with the employees before and was, thus, naïve about their specific situations.

**Table 2:** **Overview of questionnaire-based instruments and when they were administered**.

Instrument	Variable/construct	Perspective	Time of measurement		
			T0	T1	T2
Demographic questionnaire	Demographic data	P/I	X	–	–
Maslach Burnout Inventory-General Survey [MBI-GS (15)]	Emotional exhaustion, depersonalization, personal accomplishment	P/I	X	X	X
Manchester Short Assessment of Quality of Life [MANSA (16)]	Satisfaction with life as a whole and with life domains	P	X	X	X
Symptom Checklist-90-Revised [SCL-90-R (17)]	Psychological symptoms	P	X	X	X
Arbeitsbezogenes Verhaltens – und Erlebensmuster [work-related behavioral and sensational patterns] [AVEM-44 (18)]	Work-related experiences and behavior	P	X	X	X

*P, participant; P/I, participant by communicating with interviewer*.

### Sample

In order to detect a notable reduction of sickness absence days of 30%, we calculated a required total sample size of *N* = 108 per group. This calculated sample size is based on a statistical power of 0.8 and an alpha level of α = 0.05, with an assumed effect size of Cohen’s *d* = 0.3, estimated based on available IPS literature, and a total dropout rate of 20% across all measurement time points.

The sample comprised members of staff from 13 different private corporations and federal and public organizations based in northern Germany. We recruited participants who subjectively felt mentally distressed due to work-related issues or circumstances. Participation was voluntary and concealed from employers and work committees. In order to be eligible for participation, the following criteria had to be fulfilled: employee of a cooperation partner of the present study, self-report of psychological distress, voluntary participation, ability to give informed consent, and age between 18 and 67 (working age). Exclusion criteria were the need for present psychiatric inpatient treatment as well as acute suicidality.

**Figure 1:**
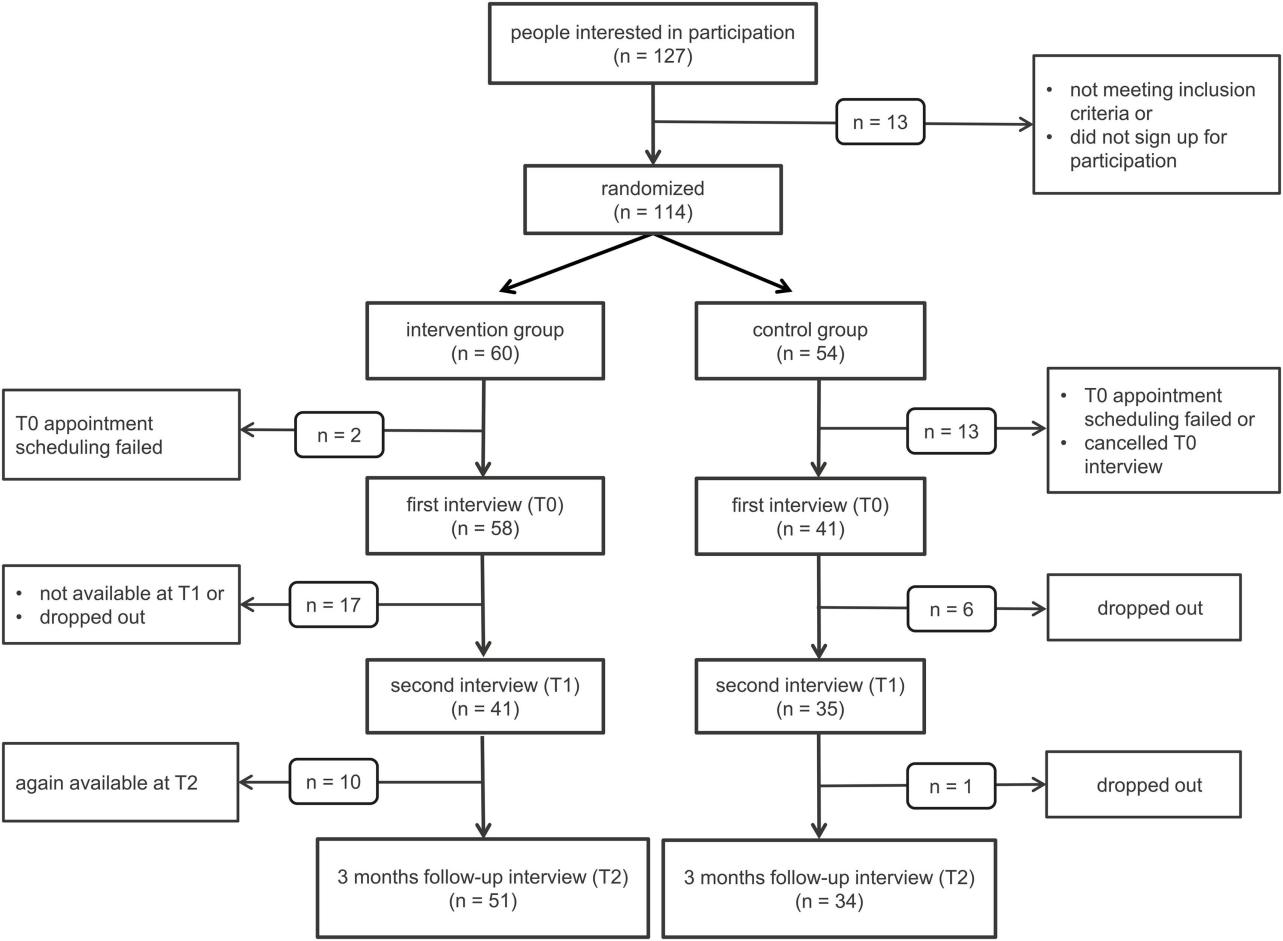
**Flow of participants**. *n*, number of participants.

Figure 1 shows the flow of participants in our randomized controlled trial and Table 3 shows the baseline data of the intervention and control group for age, gender, and the different clinical characteristics assessed with the different psychometric questionnaires we administered.

**Table 3:** **Overview and comparison of baseline data between groups**.

	Control group		Intervention group		Between group comparison		
*n*	41		58				
Gender female	63.4%		62.1%				

**T0 variable**	**mean**	**SD**	**mean**	**SD**	***t***	***df***	***p***

Age	45.10	9.03	44.17	9.47	0.49	97	0.627
Days of sickness absence (self-report)	17.76	24.30	16.16	23.58	0.33	97	0.743
AVEM – subjective importance of work	10.07	3.03	10.03	3.91	0.06	96	0.956
AVEM – professional ambition	11.88	2.85	11.64	3.52	0.35	96	0.724
AVEM – willingness to work until exhausted	13.66	3.45	12.97	3.85	0.92	97	0.360
AVEM – striving for perfection	15.71	2.17	14.39	3.17	2.45	95.80	0.016*
AVEM – distancing ability	11.18	3.49	11.81	3.53	−0.88	96	0.381
AVEM – tendency to resign (in the face of failure)	12.22	3.07	11.71	3.50	0.75	97	0.452
AVEM – proactive problem-solving	12.38	2.94	12.50	2.72	−0.22	96	0.829
AVEM – inner calm and balance	11.15	2.81	12.17	3.79	−1.53	95.48	0.129
AVEM – experience of success at work	13.33	2.81	13.54	3.19	−0.35	95	0.728
AVEM – satisfaction with life	13.71	2.97	13.97	2.96	−0.43	97	0.670
SCL mean obsessive–compulsive^a^	0.016	0.804	0.015	0.838	0.004	94	0.997
SCL – mean depression^a^	0.033	0.929	0.094	0.830	−0.333	93	0.740
Global Severity Index (total SCL score/90)^a^	−0.237	0.678	−0.292	0.899	0.313	87	0.755
Positive symptom total PST (number of SCL items with raw score >0)	44.61	17.90	44.38	19.61	0.06	97	0.953
MANSA – subjective satisfaction with life (total score)	4.91	0.79	4.76	0.82	0.88	94	0.384
MBI – cynicism	13.07	7.02	13.71	7.41	−0.43	97	0.669
MBI – emotional exhaustion	17.66	6.77	17.76	6.94	−0.07	97	0.943

*p < 0.05.

*^a^Based on logarithmized data*.

### Data Collection and Dependent Variables

Participants were recruited and data were collected on a rolling basis over a time span of 23 months. The first T0 data of participants was collected in November 2012 and the last participants provided T0 data in April 2014. The first T1 follow-up data were collected in February 2013 and the last T1 follow-up data were collected in July 2014. Regarding T2, the first follow-up data were collected in May 2013 and the last participants provided T2 follow-up data in September 2014. All data collection took place in a one-on-one, face-to-face setting, in a neutral room that was rented specifically for the purpose of the present study. The trial ended as expected after T2 data from the last recruited participant had been collected in September 2014.

The main dependent variable was the self-reported number of days the participants had been absent from work due to illness over the past 6 months (days of sickness absence). This item was included in the demographic questionnaire. Similar to Viering et al. (19), and along with other questionnaires, we also administered the following questionnaire measures to the control and intervention group to assess the participants’ mental health, life satisfaction, and work-related attitudes and behaviors before and after the study: Maslach Burnout Inventory-General Survey (MBI-GS), Manchester Short Assessment of Quality of Life (MANSA), Symptom Checklist-90-Revised (SCL-90-R), and the AVEM-44.

The MBI-GS (15, 20) is a self-report measure to assess the burnout construct with its supposed three dimensions: exhaustion [“fatigue, but without referring to people as the source of those feelings,” (21), p. 224], cynicism [“indifference or a distant attitude toward work” (21), p. 224], and professional efficacy [“social and non-social accomplishments at work” (21), p. 224]. Participants state how often they experience different work-related emotions or have work-related attitudes on a 7-point rating scale ranging from 0 = *never* to 6 = *every day*. Past research found satisfactory internal consistencies for the three MBI-GS scales across different occupational groups and nationalities [Cronbach’s alpha for the total sample ranging from 0.75 to 0.86 (22)] and acceptable factorial validity for the three-factor structure (22, 23).

The MANSA (16) is a self-report measure to assess how satisfied people are with their lives. Participants answer 12 of the 16 items on a 7-point satisfaction rating scale ranging from 1 = *couldn’t be worse* to 7 = *couldn’t be better* and four items with *yes* or *no*. The MANSA has been found to have satisfactory reliability and validity [Cronbach’s alpha = 0.74 (16)].

The SCL-90-R (17) is a self-report measure to assess general psychiatric symptomatology on nine different scales. Participants indicate the severity of symptoms they have experienced over the past 4 weeks on a 5-point rating scale ranging from 0 = *not at all* to 4 = *extremely*. The reliability of the SCL-90-R scales can be considered to be between satisfactory and very good [Cronbach’s alpha between 0.74 and 0.97 (24)]. Although the nine dimensions may not represent the best structure of the measure (25, 26), research evidence suggests good concurrent validity (25).

The AVEM-44 is a short version of the self-report AVEM questionnaire that measures the different coping styles that employees use in order to deal with their occupational workload (18). Forty-four statements build 11 scales; the scores are jointly interpreted to represent one of four distinct overall behavioral patterns (health-oriented, protection, risk of excessive demand from oneself, chronic exhaustion, and resignation). For the purposes of the present study, we did not aggregate the data to obtain the patterns but used the sum score of the 11 scales for more detailed analyses. Participants indicate their degree of agreement with different statements on 5-point rating scales ranging from: *The statement is*… 5 = *totally true* to 1 = *totally not true*. Due to high content and structural similarities between the standard and the short form, evidence for their validity apply likewise to both forms (18). Accordingly, the AVEM has been found to have satisfactory convergent, divergent, and criterion validity and Cronbach’s alpha is reported not to be lower than 0.74 for any of the 11 scales (27, 28).

### Statistical Analysis

Analyses were carried out using the SPSS statistical software version 22 (SPSS Inc, Chicago, IL, USA) according to the intention-to-treat principle. Baseline characteristics are expressed as mean and SD for continuous variables and percentages for categorical variables, respectively. The effectiveness of the job coaching intervention was evaluated with respect to the self-reported number of sickness absence days, the MBI-GS, the MANSA, the SCL-90-R, and the AVEM-44 measure.

Distributions of data were visually inspected, with several variables violating the assumption of normality. Logarithmic transformation was applied to the variables departing from normality. After the transformation, phobic anxiety, paranoid ideation, somatization, anxiety, psychoticism, hostility, interpersonal sensitivity, experience of social support, professional efficacy, and the positive symptom distress index were still skewed and, thus, excluded from further analysis.

For each of these measures, we conducted regression analyses to investigate whether the changes in scores on the different measures from T0 to T2 were predicted by group affiliation (control or intervention group). In each regression, we controlled for differences in respective T0 values, age, gender, and the professional cluster (private economy, social sector, or public service) of the company/organization. Moreover, we calculated the respective effect sizes Cohen’s *d* at T2 using the pooled SD in order to account for different group sizes (29). Effect sizes of 0.2, 0.5, and 0.8 were considered as small, medium, and large beneficial effects, respectively (30). Specifically, analyzed sample sizes at T0 were *n*_control_ = 41 and *n*_intervention_ = 58; at T1, *n*_control_ = 35 and *n*_intervention_ = 41; and at T2, *n*_control_ = 34 and *n*_intervention_ = 51. All statistical tests were performed with an alpha level of 0.05. The alpha level was not lowered due to small number of subjects. Additionally, we applied repeated measures ANOVA to investigate any group by time interaction effects for the respective outcome variables.

## Results

### Randomization Analyses

Comparisons of baseline data showed no significant group differences for gender *X*^2^ (1, *N* = 99) = 0.019, *p* > 0.05. All comparisons are shown in Table 3. There were no significant differences between the control and intervention group except for one of the AVEM-44 subscales (striving for perfection) for which the control group scored slightly higher.

### Reliability Analyses

Table 4 shows the Cronbach’s alpha coefficients for the psychometric instruments at the time points of measurement T0, T1, and T2.

**Table 4:** **Cronbach’s alpha values for the psychometric measurements at the different time points of measurement**.

Measurement	Cronbach’s alpha values		
	T0	T1	T2
MBI-GS	0.559	0.575	0.674
MANSA	0.761	0.825	0.818
SCL-90-R	0.969	0.967	0.975
AVEM-44	0.830	0.845	0.849

### Regression and Repeated Measurements ANOVA Analyses

The self-reported number of sickness absence days – our main outcome variable – was not predicted by group affiliation, *p* = 0.346, ns. Table 5 details this result and also displays the other regression results for the questionnaire measures, including corresponding effect sizes.

**Table 5:** **Regression results of questionnaire measures**.

Criterion/dependent variable	Study group affiliation as predictor						
	*B* coefficient	SE	β	*t*	*p*	Adj. *R*^2^	^a^Change in *R*^2^
Days of sickness absence (self-report)^b^	−0.335	0.352	−0.132	−0.952	0.346	0.112	0.015
AVEM – subjective importance of work	−0.922	0.532	−0.156	−1.734	0.087	0.354	0.024
AVEM – professional ambition	−0.367	0.473	−0.059	−0.776	0.440	0.535	0.003
AVEM – willingness to work until exhausted	−2.001	0.509	−0.299	−3.934	0.001**	0.537	0.086
AVEM – striving for perfection	−0.918	0.483	−0.160	−1.902	0.061	0.478	0.023
AVEM – distancing ability	1.805	0.494	0.270	3.654	0.001**	0.557	0.072
AVEM – tendency to resign (in the face of failure)	−1.248	0.479	−0.202	−2.602	0.011*	0.510	0.04
AVEM – proactive problem-solving	0.381	0.516	0.067	0.738	0.463	0.336	0.004
AVEM – inner calm and balance	0.842	0.47	0.130	1.79	0.077	0.585	0.016
AVEM – experience of success at work	1.394	0.439	0.222	3.179	0.002**	0.611	0.049
AVEM – satisfaction with life	1.316	0.44	0.212	2.989	0.004**	0.596	0.045
SCL mean obsessive–compulsive^b^	−0.206	0.153	−0.130	−1.348	0.182	0.311	0.017
SCL – mean depression^b^	−0.407	0.179	−0.220	−2.275	0.026*	0.336	0.048
Global Severity Index (Total SCL score/90)^b^	−0.221	0.172	−0.109	−1.289	0.202	0.485	0.012
Positive symptom total PST (number of SCL items with raw score > 0)	−4.918	3.093	−0.120	−1.590	0.116	0.530	0.014
MANSA – subjective satisfaction with life (total score)	0.412	0.119	0.245	3.453	0.001**	0.604	0.058
MBI – cynicism	−0.563	1.069	−0.043	−0.526	0.600	0.454	0.002
MBI – emotional exhaustion	−2.461	1.107	−0.164	−2.223	0.029*	0.549	0.027

*^a^The changes in R^2^ refer to the model in which the group affiliation as predictor was included in addition to the control variables: age, gender, the T0 value of the criterion, and professional cluster*.

*^b^Based on the logarithmic data*.

*p < 0.05.

**p < 0.01.

The regression results shown in Table 5 indicate that some changes in measure and scale values from T0 to T2 were predicted by group affiliation. Participants in the intervention group reported on average fewer depressive symptoms. Moreover, the subscales of the AVEM-44 indicated that job coaching increased the participants’ ability to distance themselves from their work as well as their experience of work-related success. Furthermore, it revealed a reduced tendency to resign in the face of failure and a decreased willingness to work until exhausted. Less depletion of emotional resources was also implied by significantly reduced MBI-GS scores of emotional exhaustion. Life satisfaction significantly increased, which is consistently indicated by the respective AVEM-44 subscale and the MANSA life satisfaction score.

We ran the same regression analyses with the participants’ data obtained at T1. This was done to investigate whether the observed pattern of results at T2 was already present at T1, right after the intervention had ended. All of the scale scores that had failed to be predicted by group affiliation at T2 were also not predicted by group affiliation at T1 (all *p* > 0.05). Moreover, the scale scores that were significantly influenced by the job coaching intervention at T2 did not prove to be already significant at T1, except for the AVEM-44 subscale of life satisfaction. For this subscale, being in the intervention group predicted a significant increase in life satisfaction scores at T1 with *b* = 0.96, *t*(68) = 2.08, *p* = 0.041.

Effect sizes at T2 indicated that the job coaching had the greatest absolute significant effect on the willingness to work until exhausted (*d* = 0.82) and on participants’ distancing ability (*d* = −0.69). The smallest absolute significant effect was observed for emotional exhaustion (*d* = 0.23).^1^

In addition to the performed regression analyses, we also checked for any time by group interaction effects using a repeated measures ANOVA. Results mirrored the aforementioned pattern or results of the regression analyses, that is, which effects turned out to be significant and which not, did not change.

## Discussion

With the present trial, we evaluated the effectiveness of job coaching as an on-the-job intervention for mentally distressed employees. The application of one-on-one job coaching represents a specific form of supported employment and has been derived from the concept of IPS (4). Contributing to the body of literature on preventive job coaching, we conducted a randomized controlled trial with the self-reported number of sickness absence days as the main outcome criterion. In addition, our study included several questionnaire measures to assess clinical symptoms, burnout indicators, and participants’ satisfaction with different domains of their life, such as their job and their physical and mental health. Both the intervention as well as the control group completed these measures before the study started (T0), after ~3 months (T1), which represented the end of the job coaching sessions for the intervention group, and at a 3-month follow-up (T2).

Regarding self-reported sickness absence days, no significant effect of job coaching was detected when the two groups were compared (at neither T1 nor T2). However, our analyses of the questionnaire measures revealed that compared with the control group the intervention group significantly benefited from the job coaching. Job coaching reduced symptoms of depression as well as emotional exhaustion and improved participants’ distancing ability, their experience of success at work, the tendency to resign, and the satisfaction with their lives. These effects showed for data collected at the 3-month follow-up interview but not directly at the end of the intervention. Immediately after the job coaching had ended, at T1, only the AVEM-44 subscale scores of life satisfaction had already significantly increased.

Generally, our results are consistent with related research that evaluated IPS interventions (4). IPS has been found to be an effective means to reintegrate people with mental disorders into the job market (5). Compared with IPS, the intervention in our trial addressed experienced mental distress at work in an early stage that might have already negatively affected the employees’ work performance. To some extent mirroring our pattern of results, evidence for beneficial effects of job coaching interventions was provided by Duijts et al. (12). These authors also did not find a reduction of “self-reported sickness absence due to psychosocial health complaints” [(12), p. 770] through job coaching, but they reported, however, a reduction of the objective number of sickness absence days in a period from baseline to an 8-month follow-up. Moreover, they also found better self-rated health, less burnout exhaustion and fewer depressive reactions for participants who received job coaching between baseline and 1-year follow-up (12).

Still, there are also more equivocal findings for the effect of coaching interventions on depression, stress levels, or anxiety. While Gyllensten and Palmer (9) reported a significant positive effect on stress and anxiety but not on depression, Grant et al. (8) could not find an effect on anxiety and reported ambiguous relationships for depression and stress. However, meta-analytical findings have recently provided further evidence for the effectiveness of coaching in organizational contexts (11). These findings support the notion that job coaching can improve employees’ well-being, coping abilities, work attitudes, and goal-directed self-regulation. The results of the present research are consistent with this meta-analytical evidence (11) and further corroborate the validity of job coaching as a means to ameliorate employees’ psychological conditions.

Although we did not observe a decline in the self-reported days of sickness absence at a 3-month follow-up between the groups, it could be speculated that this benefit may have a delayed onset. Psychological improvements may need to take effect first to translate into reduced sickness absence. A delayed onset might occur because the psychological effects of job coaching were7 not directly observed at the end of the intervention at T1 but they were present 3 months after the intervention had ended. This fact might indicate that tackling mental distress to create positive behavioral outcomes via job coaching is at least a mid-term process that requires time before the full benefits may become evident. This assumption is, however, somewhat challenged by findings reported by McGonagle et al. (10) who observed coaching benefits immediately at the end of the coaching intervention and also at a 12-week follow-up. Yet, the overall findings reported by McGonagle et al. (10), that is, improved personal well-being but no improved job well-being, are in line with the present results. Still, more research is necessary to further determine the factors that influence and drive the timely dimension in terms of the onset of positive effects of job coaching. Similarly, the generalizability and applicability of job coaching interventions to different work environments and situations should be subject to further research.

Our results still show that job coaching has beneficial outcomes. The effects of improved satisfaction as well as decreased emotional exhaustion and fewer symptoms of depression that we found can contribute to maintaining the work performance of employees. However, it should be noted that job coaching is no therapeutic intervention and is not a substitute for it. When a need for therapy is recognized, the job coach may act as mediator to triangulate between the employee’s supervisor and therapist. This triangulating function adds to the value of job coaching in companies and organizations. It has to be taken into account that disclosure of mental distress or a mental disorder has to be considered deliberately (31). However, a study by Allott et al. (32) revealed that IPS can be done with different disclosure preferences and that disclosure does not predict vocational outcome in persons with recent onset psychosis. If sickness absence can be reduced and jobs can be maintained through job coaching, these effects should also translate into lowered costs and ultimately higher economic returns for the organization. Thus, the introduction of job coaching in organizations would literally pay off.

## Limitations

In spite of the promising results found for job coaching as a tool to support mentally distressed employees, some limitations of the present study need to be acknowledged. First, the measurement of the main outcome criterion of sickness absence days needs to be discussed. Participants had to recall their sickness absence days during the past 6 months. Thus, this measure might be inaccurate or prone to recall bias (33, 34). Moreover, it would have been better to ask participants about absence within a shorter timeframe (e.g., 30 or 60 days) as data were collected in 90 days intervals but they had to recall their absence in the past 6 months. Thus, there is an overlap of 3 months between the recalled time periods of sickness absence and the chronological phases of the study. That is, T1 data, collected at the end of the 3 months coaching intervention, include the 3 months prior to the baseline measurement point (T0), since we asked participants to recall their absence in the last 6 months. Similarly, the T2 follow-up data, collected 3 months after the coaching, include the 3 months coaching period. However, T0 data and T2 data, which we investigated with the performed analyses, do not include overlapping time periods since T2 data were collected 6 months after T0. Still, it is important for future studies to address these aspects in the measurement strategy, for example, by only recording data of the intervention period and the follow-up period (e.g., 3 months each) with no overlap, and compare it to baseline data that cover the same period of time prior to the start of the intervention.

Second, the duration of coaching in the present trial may be considered to be rather short compared with, for example, the EQOLISE, the ZInEP or the ZhEPP trials (5, 19, 35). This brevity of coaching may be a reason why we only found partial beneficial effects in our data. Furthermore, we employed only a 3-month follow-up assessment. The follow-up period should be extended since sustainability is an issue in IPS interventions (36). An extended follow-up [see, for example, Ref. (12, 37)] would allow evaluating the sustainability of the intervention and provide a more accurate judgment of its long-term effects.

Third, it needs to be highlighted that the reported internal consistencies of the MBI-GS scale are quite low, with values ranging between 0.559 (T0) and 0.674 (T2). We could not identify any reasons why these coefficients are low, since we administered the standard version of the MBI-GS scale. Although it is widely stated that alpha coefficients should amount to at least 0.70 or greater in order to be regarded as acceptable, Schmitt (38) points out that even if a measure has a lower reliability, this does not necessarily have to be a drawback to its use, if it, for example, covers the construct of interest well. Still, in light of the low internal consistencies of the MBI-GS in the present study, our results for this measure have to be interpreted with caution.

## Conclusion

We have shown that job coaching for mentally distressed employees improves several personal domains that contribute to re-establish the employees’ full capacities to work. Although we did not find significantly reduced days of sickness absence when comparing the intervention and control group, job coaching seems to be a viable tool to benefit the psychological conditions of employees. If the re-establishment and maintenance of the employees’ capacity to work translates into reduced sickness absence in the long run, the introduction of a job coaching program may create a win–win situation since reduced sickness absence results in lower costs incurred by the organization. Job coaching can, therefore, be regarded as an effective intervention tool for companies’ health care management systems that benefits both the employees and the organization.

## Author Contributions

N-TT, VS, and JM conducted the data processing. N-TT and WK drafted the manuscript. WK and WR conceptualized and conducted the study as principal investigators. WK, SH, and WR supervised the intervention and the data acquisition. All authors read and approved the final version of the manuscript.

## Conflict of Interest Statement

The present research was conducted in the absence of any commercial or financial relationships that could be construed as a potential conflict of interest. The handling editor declared a shared affiliation, though no other collaboration, with the authors WR and WK and states that the process nevertheless met the standards of a fair and objective review.

## Footnotes

^1^Please note that the effect sizes were calculated by subtracting the mean values obtained at T2 from the mean values obtained at T0. This means, for example, that the negative effect size of participant’s distancing ability (*d* = −0.69) indicates an *increased* distancing ability at T2. Vice versa, the positive effect size of, for example, emotional exhaustion (*d* = 0.23) indicates that the mean emotional exhaustion was found to be *lower* at T2 than it had been at T0.
